# New genes on the block: Neofunctionalization of tandem duplicate genes with putative new functions in Arabidopsis

**DOI:** 10.1093/plphys/kiad271

**Published:** 2023-05-09

**Authors:** José Manuel Ugalde, Henryk Straube

**Affiliations:** Assistant Features Editor, Plant Physiology, American Society of Plant Biologists, USA; INRES-Chemical Signalling, University of Bonn, Friedrich-Ebert-Allee 144, 53113 Bonn, Germany; Assistant Features Editor, Plant Physiology, American Society of Plant Biologists, USA; Faculty of Science, Department of Plant and Environmental Sciences, Section for Plant Biochemistry, University of Copenhagen, Copenhagen 20855-2768, Denmark

Gene duplication is a common phenomenon during evolution, notably more prevalent in plants than in other organisms. Gene duplication is considered a driving force for evolutionary innovation because newly duplicated genes can acquire new functions or become differentially expressed to the original gene copy. These processes are referred to as neofunctionalization. Duplicated genes are useful for studying how an organism's traits become specialized over time during evolution because genes that have recently been duplicated should have undergone fewer changes between them, making them a good model to observe the process of functionalization (Huang et al. 2022). However, there is a gap in the capacity to study phenotypic functionalization during plant evolution because a critical mass of experiments, sequencing data, and phenotyping information is still missing.

**Figure 1.**
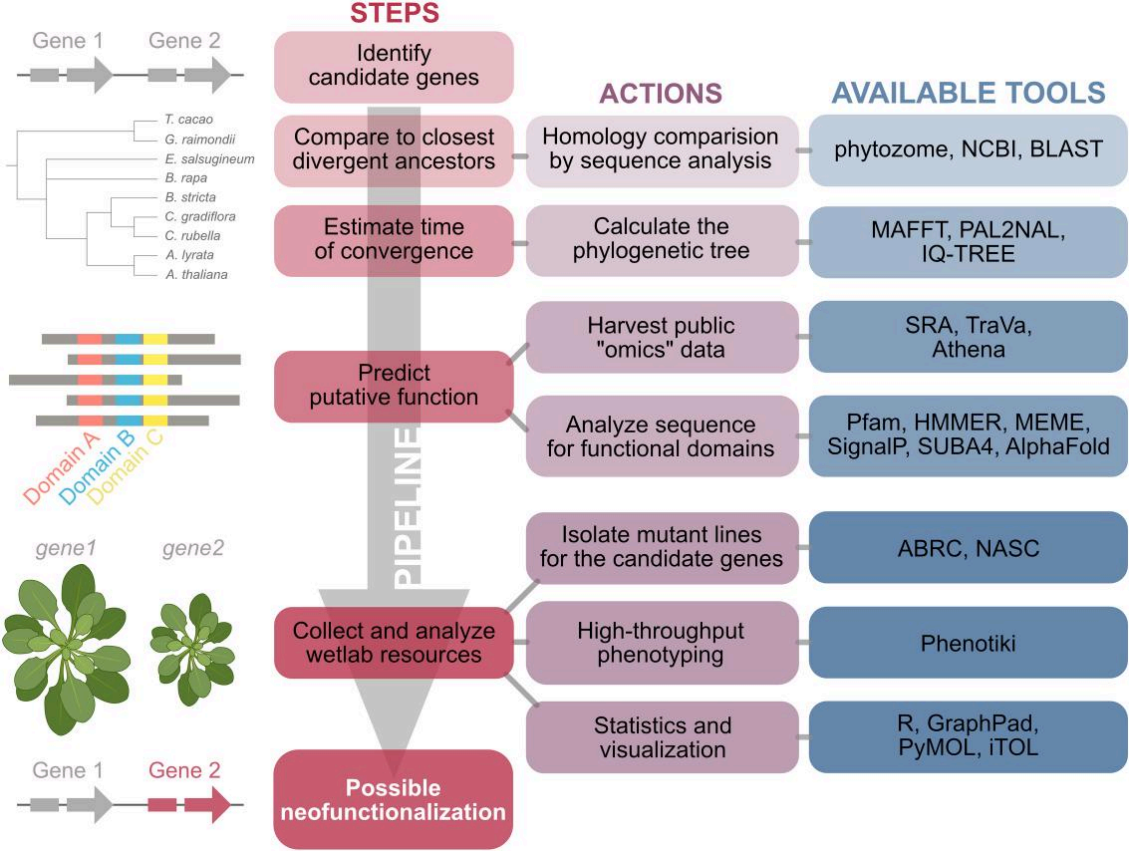
Pipeline strategy to establish possible neofunctionalization events. Once the candidate genes have been identified, the analysis can be carried out step by step (left boxes). An action is indicated for each step (middle boxes), for which several tools can be used. Here, we list the tools Tao et al. used to complete each step of the analysis (right boxes). Briefly, the sequence of the candidate genes should be compared to the closest divergent ancestors to establish the estimated time of convergence. Putative functions can be attributed to the candidate genes by mining the public expression data and analyzing if their sequence has annotated functional domains. Knowing the putative role of these proteins, a biochemical characterization needs to be performed on them. Finally, deficient lines in the candidate genes should be phenotyped to establish the functional relevance of each gene in the plant.

In this issue of *Plant Physiology*, Tao et al. (2023) provide a comprehensive pipeline for the analysis of putative neofunctionalized genes (Figure 1). The authors studied the highly similar genes AT5G12950 and AT5G12960 identified in a previous study (Wang et al. 2016) and annotated them as putative β-L-arabinofuranosidase 1 and 2 (*PAF1* and *PAF2*). In plants, algae, and some bacteria, the aldopentose L-Arabinose (L-Ara) is a highly abundant monosaccharide that accounts for 5% to 10% of cell wall saccharides in Arabidopsis (*Arabidopsis thaliana*) or rice (*Oryza sativa*) (Kotake et al. 2016). L-Ara exists as 2 tautomers, L-arabinofuranose (L-Araf) and L-arabinopyranose (L-Arap). It is a crucial component of various compounds, such as rhamnogalacturonan, xylans, xyloglucans, glycosylated proteins, as well as specialized metabolites like flavonoids and saponins (Kotake et al. 2016; Mariette et al. 2021).

Although L-Ara*p* is the most abundant form of L-Ara in biopolymers, it is mostly incorporated into its target molecules as Uridine-5'-diphospho-1-Ara*f* (UDP-l-Araf; Mariette et al. 2021). UDP-l-Ara*f* is either synthesized via the de novo biosynthesis pathway from Uridine-5′-diphosphoglucose and glucuronic acid or by the salvage pathway that recycles L-Ara derived from cell wall degradation (Mariette et al. 2021). PAF1 and 2 would catalyze the initial reaction of the L-Ara salvage pathway, by releasing L-Ara from cell wall components (Tao et al. 2023). The pipeline presented by Tao et al. (Figure 1) for the analysis of putative neofunctionalized genes represents a useful starting point toward a better understanding of how neofunctionalization contributes to the evolution of L-Ara salvage pathways.

Tao and colleagues established that the 2 candidate genes are derived from a tandem gene duplication 16 million years ago by performing phylogenetic analysis, syntenic analysis, and comparing the intron/exon structure of the genes and their homologs in closely related species. Analyzing the protein sequence of all identified potential PAFs using different bioinformatic tools like the Protein Families database and the hidden Markov model statistical model (www.hmmer.org), the authors identified that the sequences contained a glycosidase domain, GH127, that had previously only been described in bacteria (Nakamura et al. 2018). A potential extracellular localization was estimated by using ChloroP and SUBA4, indicated by an N-terminal signal peptide.

The researchers then reanalyzed published transcriptomic and proteomic data to explore if a differential expression between the *PAF* genes could add to the evidence that at least 1 of these genes went through a potential neofunctionalization. Interestingly, the expression pattern and protein abundance differed between *PAF1* and *PAF2*. *PAF1* showed an even expression pattern in several different tissues, whereas *PAF2* was mostly expressed in pollen-related tissues. This result is in line with a coexpression with *ARABINOKINASE 1* and *UDP-SUGAR PYROPHOSPHORYLASE*, 2 other genes involved in the L-Ara salvage pathway. The coexpression points to a specific role of *PAF2* in pollen tissues, whereas *PAF1* likely possesses a broader function. The authors furthermore studied the promotor region of *PAF1* and *PAF2* and concluded that variance in *cis*-regulatory elements could be explanatory for the difference in expression and functionality. To estimate the impact of both genes on the phenotype, the investigators used the automated image-based phenotyping framework Phenotiki and discovered slight phenotypic alterations in *paf1* and *paf2* compared with wild-type plants (Figure 1).

In summary, Tao et al. collected evidence indicating neofunctionalization between the tandem genes PAF1 and PAF2, the latter a newer version expressed in more plant tissues. This study compiles a set of tools that allow further understanding of how gene duplication is linked to new functions in plants. However, further biochemical evidence is needed to confirm these findings and provide more insight into the functions of these genes. A better understanding of the L-Ara pathways is urgently needed because the response of plants to climate change-linked effects like development, structure, and environmental adaption are in part determined by the cell wall.

In the model plant Arabidopsis, 85% of all genes are homologous to at least another gene, and depending on the plant species, 5% to 20% of genes are estimated to be duplicated in tandem (Cannon et al. 2004; Jayakodi et al. 2023). In Arabidopsis, for example, some of the gene families with a higher number of tandem duplicates are related to pathogen defense (GERMIN-LIKE, the major latex protein-related) or coding for multifunctional glutathione transferases (Cannon et al. 2004). In faba bean (*Vicia faba*), genes coding for polyphenol oxidases (*PPO*s) are highly similar and may have arisen by gene duplication. Comparative genomic, transcriptomic, and phylogenetic data suggest that mostly 1 of the *PPO*s is responsible for the hilum color of faba bean seeds by differential transcriptional regulation (Jayakodi et al. 2023). The toolbox presented here by Tao et al. is a precedent for a pipeline strategy to analyze tandem duplicated genes in plants, shedding light on possible unseen neofunctionalization that has occurred in duplicated gene families.
